# Trends in and Determinants of Loss to Follow Up and Early Mortality in a Rapid Expansion of the Antiretroviral Treatment Program in Vietnam: Findings from 13 Outpatient Clinics

**DOI:** 10.1371/journal.pone.0073181

**Published:** 2013-09-16

**Authors:** Dam Anh Tran, Anh Duc Ngo, Anthony Shakeshaft, David P. Wilson, Christopher Doran, Lei Zhang

**Affiliations:** 1 Kirby Institute, The University of New South Wales, Sydney, New South Wales, Australia; 2 National Drug Alcohol Research Centre, The University of New South Wales, Sydney, New South Wales, Australia; 3 School of Population Health, The University of South Australia, Adelaide, South Australia, Australia; 4 Hunter Medical Research Centre, The University of Newcastle, Newcastle, Australia; Centers for Disease Control and Prevention, United States of America

## Abstract

**Background:**

This study aims to describe the trends in and determinants of six month mortality and loss to follow up (LTFU) during 2005–2009 in 13 outpatient clinics in Vietnam.

**Method:**

Data were obtained from clinical records of 3,449 Vietnamese HIV/AIDS patients aged 18 years or older who initiated ART between 1 January 2005 and 31 December 2009. Mantel-Haenszel chi-square test, log rank test were conducted to examine the trends of baseline characteristics, six month mortality and LTFU. Cox proportional hazards regression models were performed to compute hazard ratio (HR) and 95% Confidence Interval (CI).

**Results:**

Though there was a declining trend, the incidence of six month mortality and LTFU remained as high as 6% and 15%, respectively. Characteristics associated with six month mortality were gender (HR females versus males 0.54, 95%CI: 0.34–0.85), years of initiation (HR 2009 versus 2005 0.54, 95%CI: 0.41–0.80), low baseline CD4 (HR 350–500 cells/mm^3^ versus <50 cells/mm^3^ 0.26, 95%CI: 0.18–0.52), low baseline BMI (one unit increase: HR 0.96, 95%CI: 0.94–0.97), co-infection with TB (HR 1.61, 95%CI: 1.46–1.95), history of injecting drugs (HR 1.58, 95%CI: 1.31–1.78). Characteristics associated with LTFU were younger age (one year younger: HR 0.97, 95%CI: 0.95–0.98), males (HR females versus males 0.82, 95%CI: 0.63–0.95), and poor adherence (HR 0.55, 95%CI: 0.13–0.87).

**Conclusions:**

To reduce early mortality, special attention is required to ensure timely access to ART services, particularly for patients at higher risk. Patients at risk for LTFU after ART initiation should be targeted through enhancing treatment counselling and improving patient tracing system at ART clinics.

## Introduction

Loss to follow up (LTFU) and mortality after initiation of antiretroviral therapy (ART) are commonly used outcomes to evaluate the effectiveness of ART and to identify opportunities for program improvement [1]–[3]. Both the socio-demographic and clinical characteristics of patients at ART initiation can predict the rates of LTFU and mortality [1], [4]. For patient characteristics, low baseline CD4 count and TB or other co-infections have been consistently associated with early mortality [1], [5], [6], while age and baseline BMI have been associated with early mortality in Myanmar [7] and Johannesburg [8], but not in South Africa [1], [8]–[10], and being male is positively associated with LTFU in South Africa [11] and Tanzania [12] but not in Lesotho [13].

Despite significant expansion and increased coverage of HIV care and treatment in Vietnam, high rates of LTFU (e.g., 12% in 2009 nationwide) have been reported [14], with many deaths being recorded in the first year following ART initiation (e.g., first year mortality rate was 8.7 per 100 person-years) [15]. Three studies have examined LTFU and mortality rates in patients receiving ART. One study conducted in 27 adult and 4 paediatric clinics (4,531 adults and 313 children) reported that while the 12 month LTFU rate in patients initiating ART in 2007–2009 was 18.8% [14], the rate was significantly lower in tertiary-level clinics compared with secondary-level clinics, but higher in clinics serving less than 100 patients. Another study involving 640 patients in 13 outpatient clinics in Quang Ninh province identified a number of significant predictors of AIDS-related mortality within 15 months of initiation to ART: being older, initiating ART at a relatively late clinical stage (i.e., stage III or IV), having a low baseline BMI and a low baseline CD4 cell count [16]. This study also found that being male and having a low BMI or CD4 cell count at baseline were significantly associated with early mortality [17]. To date, however, no studies have identified patient characteristics associated with LTFU or considered mortality in the first six months as an outcome. Furthermore, having a short follow up (≤3 years), these studies could not assess the trend of these two important outcomes.

LTFU and early mortality present key challenges in the implementation of ART programmes. High rates of early mortality, in particular, can reflect a high level of treatment failure, drug resistance, or poor adherence to treatment regimens [5], [6], [8]. Similarly, a high level of LTFU can be a marker of deficiencies in treatment counselling and patient tracing system at the ART clinic [8], [18], [19]. Understanding trends in, and determinants of, LTFU and early mortality, therefore, is important in optimising ART patient outcomes, especially in Vietnam where there has been a recent and rapid up-scaling of ART services. This study aimed to: (i) describe the trends in early mortality and LTFU in HIV patients who initiated the treatment within the first five years of the country's national ART programme (2005–2009); (ii) identify patient demographic and clinical characteristics that predict LTFU and six month mortality.

## Methods

### Study design and sample size

A prospective study design was used. The complete description of cohort participants and the selection of clinics has been previously published [17]. Briefly, the study sample contained 3,449 adults (≥18 years) who initiated ART from 1st of January, 2005 to 31st of December, 2009 in 13 outpatient clinics, located in six provinces across different geographic regions in Vietnam. Ethical approval was granted by the Hanoi School of Public Health Ethical Review Board before data collection was conducted: Participants provided their written informed consent to participate in this study. The consent forms were provided to the patients, and signatures were obtained. This consent procedure was approved by the Hanoi School of Public Health Ethical Review Board.

### Data collection and measurements

In the present analysis, outcome measures were deaths occurring in the first six months of ART and patients who were identified as LTFU during the entire follow up period. Patients were defined as LTFU based on the patient records: if they had started ART and were absent from the clinic for more than three months and did not return to the clinics by the end of the study period (30 November 2010).

Independent variables included: baseline age (expressed in years) and gender; transmission routes (injecting drug use [IDU], unsafe sex [USS]); baseline clinical characteristics (body mass index [BMI], CD4 cell counts, WHO clinical stage, TB co-infection, opportunistic infection [OI], Cotrimoxazol [CTX] use); adherence to treatment every six months following ART initiation; and the (calendar) year of ART initiation. Adherence was designated as “Good” if patient had >95% self-reported adherence rate in the previous month, and “Poor” if the patient had <95%.

### Statistical analysis

Descriptive statistics were performed to derive means and standard deviations for continuous variables (age, baseline BMI) and to provide frequency distributions for categorical variables (proportion of WHO stage IV, proportion of IDU, USS, gender, baseline OI, baseline CTX, baseline TB, treatment adherence). Difference in trend of proportion of baseline WHO stage, baseline OI, baseline CTX, baseline TB by initiation years were assessed by Mantel-Haenszel chi-square test. Differences in trend of six month mortality and LTFU by initiation years were assessed using log rank test. The relationships between baseline characteristics and outcomes were tested using proportional hazards regression models. Crude and adjusted hazard ratios (HR) and 95% Confidence Intervals (CI) were reported. All analyses were performed using SAS (version 9.2).

The analysis began with univariate analysis to identify characteristics that were likely to be associated with the outcome (*p*<0.25), and these variables were subsequently fitted into the initial multivariate Cox regression models. Among variables that were not significantly associated with the outcome (*p*>0.05), the one that was least significant (highest *p* value) was removed and the model refitted with the remaining variables. This procedure was repeated in a backward stepwise manner. In the final model, only variables that were significantly associated with the outcome (*p*≤0.05) were retained.

## Results

### Patient characteristics

The current analysis involved 3,449 patients, 75% being males, who initiated ART between 1^st^ of January 2005 and 31^st^ December 2009. By the end of the study, there were a total of 18,751 person-years of follow-up (median 1.38 years, interquartile range [IQR] 0.75–2.47). The mean age at baseline was 30 years old, 65% were infected through injecting drugs. After five years of follow up, 198 (5.7%) patients have died within first six months on ART, 530 (15.4%) patients were lost to follow up. The descriptive information of study participants in the cohort, the patients died in the first six months of ART, and the LTFU are presented in Table 1.

**Table 1:** Summary of baseline characteristics of PLHIV in 13 outpatient clinics in Vietnam [N = 3,449].

Characteristics		Patients who died within six months of ART	Patients who were lost to follow up during study period	Total PLHIV
		N = 198n (%)	N = 530n (%)	N = 3,449n (%)
Baseline age years mean (SD)		29.5 (5.8)	29.2 (6.0)	30.3 (6.6)
Gender	Male	141 (71.2)	426 (80.4)	2,573 (74.6)
	Female	57 (28.8)	104 (19.6)	876 (25.4)
Baseline CD4	<50	99 (50.0)	305 (57.5)	1,538 (44.6)
(cells/mm^3^)	50–100	72 (36.4)	82 (15.5)	503 (14.6)
	100–200	20 (10.1)	87 (16.4)	649 (18.8)
	200–350	1 (0.5)	38 (7.2)	320 (9.3)
	350–500	0 (0.0)	10 (1.9)	53 (1.5)
	>500	0 (0.0)	4 (0.8)	43 (1.2)
	Missing	6 (3.0)	4 (0.8)	343 (9.9)
ART initiation year	2005	62 (31.3)	23 (4.3)	105 (3.0)
	2006	47 (23.7)	123 (23.2)	497 (14.4)
	2007	38 (19.2)	128 (24.2)	891 (25.8)
	2008	26 (13.1)	151 (28.5)	966 (28.0)
	2009	14 (7.1)	89 (16.8)	843 (24.4)
	Missing	11 (5.6)	16 (3.0)	147 (4.3)
History of IDU^a^	Yes	152 (76.8)	380 (71.7)	2,250 (65.2)
	No	43 (21.7)	98 (18.5)	937 (27.2)
	Missing	3 (1.5)	52 (9.8)	262 (7.6)
History of USS^b^	Yes	78 (39.4)	124 (23.4)	1,070 (31.0)
	No	107 (54.0)	358 (67.5)	2,270 (65.8)
	Missing	13 (6.6)	48 (9.1)	109 (3.2)
Baseline BMI^c^ kg/m^2^		13.92 (7.3)	16.33 (6.1)	16.63 (8.4)
Baseline OI^d^	Yes	128 (64.6)	364 (68.7)	1,962 (56.9)
	No	49 (24.7)	134 (25.3)	1,191 (34.5)
	Missing	21 (10.6)	32 (6.0)	296 (8.6)
Baseline CTX^e^	Yes	107 (54.0)	372 (70.2)	2,115 (61.3)
	No	82 (41.5)	124 (23.4)	916 (26.6)
	Missing	9 (4.5)	34 (6.4)	418 (12.1)
Baseline TB^f^	Yes	150 (75.8)	398 (75.1)	1,121 (32.5)
	No	41 (20.7)	113 (21.3)	2,091 (60.6)
	Missing	7 (3.5)	19 (3.6)	237 (6.9)
Baseline WHO stage	Stage I	8 (4.0)	8 (1.5)	76 (2.2)
	Stage II	24 (12.1)	54 (10.2)	434 (12.6)
	Stage III	38 (19.2)	74 (14.0)	692 (20.1)
	Stage IV	109 (55.1)	378 (71.3)	1,919(55.6)
	Missing	19 (9.6)	16 (3.0)	328 (9.5)
Adherence	Poor	81 (40.9)	425 (80.2)	975 (28.3)
	Good	105 (53.0)	78 (14.7)	2,145 (62.2)
	Missing	12 (6.1)	27 (5.1)	329 (9.5)

a *: IDU: Injecting drug user, ^b^: USS: Unsafe sex, ^c^: BMI: Body mass index, ^d^: OI: Opportunistic infection, ^e^: CTX:*

*Cotrimoxazol use, ^f^: TB: Tuberculosis*.

### Trends in patient clinical characteristics and outcomes

**Figure 1:**
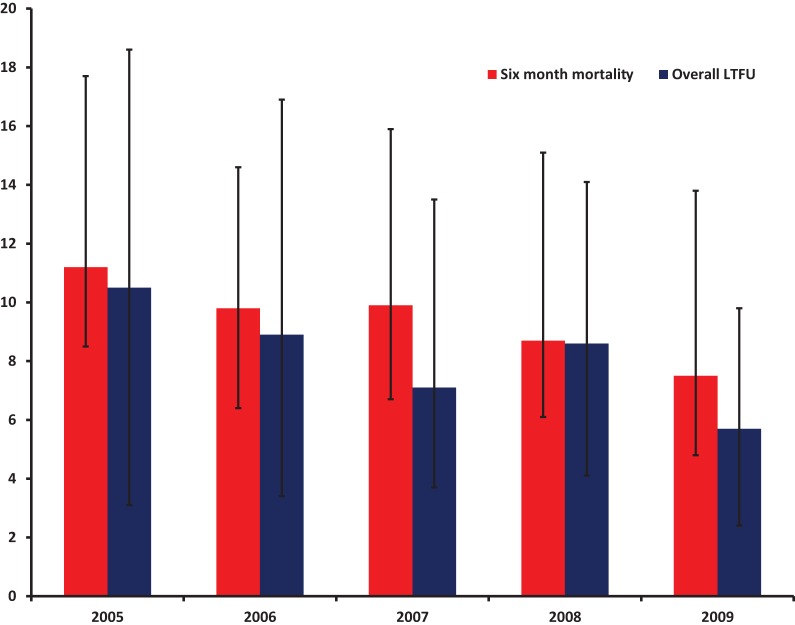
Trends in six month Mortality and LTFU by calendar years in 13 outpatient clinics in Vietnam (N = 3,449).

As summarised in Table 2, overall, numbers of initiation patients increased progressively by calendar year from 105 in 2005 to 966 in 2008. In each year from 2005 (when the ART program in Vietnam was initiated), patients were enrolled at a less advanced clinical stage and with higher baseline CD4 cell counts. Specifically, baseline CD4 cell counts increased from 59 cells/mm^3^ (SD: 45) in 2005 to 101 (SD: 86) cells/mm^3^ in 2009 (simple linear regression p = 0.001). The proportion of patients at Stage IV decreased from 61% in 2005 to 39% in 2009 (Mantel-Haenszel chi-square test p = 0.01). However, there was no significant trend in the proportion of patients with baseline OI (Mantel-Haenszel p = 0.43), TB co-infection (Mantel-Haenszel p = 0.51), and baseline CTX use (Mantel-Haenszel p = 0.73). As presented in Figure 1, between 2005 and 2009, the six month mortality rate declined from 11.2 to 7.5 per 100 person years (log rank test p = 0.001), and the overall LTFU decreased from 16.2 to 8.9 per 100 person years (log rank test p = 0.001).

**Table 2:** Summary of baseline characteristics, six

Characteristics		Year 2005 (n = 105)	Year 2006 (n = 497)	Year 2007 (n = 891)	Year 2008 (n = 966)	Year 2009 (n = 843)	p-value^a^
Baseline age years mean (SD)		28.8 (6.5)	29.3 (6.2)	30.1 (6.3)	30.9 (6.8)	30.5 (6.9)	0.37
Gender	Male	87 (82.9)	380 (76.5)	680 (76.3)	688 (71.2)	613 (72.7)	0.25
	Female	18 (17.1)	117 (23.5)	211 (23.7)	278 (28.8)	230 (27.3)	
Baseline CD4 (cells/mm^3^)		59 (45)	87 (78)	91 (80)	94 (86)	101 (86)	0.00
Mean (SD)							
History of IDU^c^	Yes	76 (72.6)	324 (65.2)	655 (73.5)	679 (70.3)	542 (64.3)	0.57
	No	21 (19.8)	128 (25.7)	202(22.7)	244 (25.2)	263 (31.2)	
	Missing	8 (7.6)	45 (9.1)	34 (3.8)	43 (4.5)	38 (4.5)	
History of USS^d^	Yes	41 (39.2)	213 (42.9)	259 (51.8)	311 (51.3)	314 (39.0)	0.32
	No	55 (52.2)	256 (51.5)	605 (67.9)	626 (64.8)	507 (60.1)	
	Missing	9 (8.6)	28 (5.6)	27 (3.0)	29 (3.0)	22 (2.6)	
Baseline BMI^e^ kg/m^2^		16.0 (6.9)	16.9 (6.8)	17.2 (5.9)	18.1 (5.4)	18.2 (7.11)	0.26
Baseline OI^f^	Yes	54 (51.2)	248 (49.9)	462 (51.8)	496 (51.3)	329 (39.0)	0.43
	No	40 (38.3)	230 (46.3)	401 (45.1)	436 (45.2)	486 (57.7)	
	Missing	11 (10.5)	19 (3.8)	28 (3.1)	34 (3.5)	28 (3.3)	
Baseline CTX^g^	Yes	66 (63.0)	388 (78.0)	719 (80.7)	820 (84.9)	721 (85.5)	0.73
	No	27 (25.6)	73 (14.8)	130 (14.6)	105 (10.9)	90 (10.7)	
	Missing	12 (11.4)	36 (7.2)	42 (4.7)	41 (4.2)	32 (3.8)	
Baseline TB^h^	Yes	38 (36.0)	169 (34.0)	268 (30.1)	298 (30.9)	257 (30.5)	0.51
	No	53 (50.7)	296 (59.6)	575 (64.5)	625 (64.6)	548 (65.0)	
	Missing	14 (13.3)	32 (6.4)	48 (5.4)	43 (4.5)	38 (4.5)	
Baseline WHO stage	Stage IV	64 (61.0)	268 (54.0)	407 (45.7)	396 (41.0)	330 (39.1)	0.01
	Other stage	29 (27.6)	191 (38.4)	445 (49.9)	533 (55.2)	480 (57.0)	
	Missing	12 (11.4)	38 (7.6)	39 (4.4)	37 (3.8)	33 (3.9)	
Adherence	Poor	32 (30.0)	139 (28.0)	276 (31.0)	213 (22.0)	143 (17.0)	0.28
	Good	63 (59.5)	327 (65.8)	580 (65.1)	714 (74.0)	666 (79.0)	
	Missing	11 (10.5)	31 (6.2)	35 (3.9)	39 (4.0)	34 (4.0)	
Six month mortality^b^		11.2 (8.5–13.7)	9.9 (6.7–10.7)	9.8 (6.4–11.5)	8.7 (6.1–10.4)	7.5 (4.8–9.6)	<0.01
Overall LTFU^b^		16.2 (10.3–19.4)	14.1 (11.1–18.7)	13.8 (8.6–14.9)	9.8 (5.9–11.0)	7.1 (5.3–10.5)	<0.01

a *: p value from simple linear regression tests were presented for baseline CD4, baseline age, and baseline BMI; p value from Mantel-Haenszel chi-square tests were presented for gender, history of IDU, history of USS, baseline OI, baseline CTX, baseline TB, baseline WHO stage, and adherence; p value from log rank tests were presented for six month mortality and overall LTFU*.

b *: Six month mortality and overall LTFU were presented as rates per 100 person years with confidence interval*.

c *: IDU: Injecting drug user, ^d^: USS: Unsafe sex, ^e^: BMI: Body mass index, ^f^: OI: Opportunistic infection, ^g^: CTX:*

*Cotrimoxazol use, ^h^: TB: Tuberculosis*.

**Table:** Table 3. Cox Proportional Hazards Model of 6 month Mortality and LTFU by baseline characteristics and years of ART initiation in PLHIV in 13 outpatient clinics in Vietnam.

Characteristics			6 month mortality (n = 2,903)		P value	LTFU during 2005–2009 (n = 2,988)		p value
			Univariate, HR (95%CI)	Multivariate, HR (95%CI)		Univariate, HR (95%CI)	Multivariate, HR (95%CI)	
Baseline age^g^			0.99 (0.97–1.01)	1.00 (0.98–1.02)		0.97 (0.95–0.98)	0.97 (0.95–0.98)	
Gender	Male		1.00	1.00		1.00	1.00	
	Female		**0.40 (0.29–0.57)**	**0.54 (0.34–0.85)**		**0.59 (0.41–0.87)**	**0.82 (0.63–0.95)**	
Baseline CD4 (cells/mm^3^)	<50		1.00	1.00		1.00	1.00	
	50–100		**0.70 (0.38–0.82)**	**0.53 (0.30–0.91)**		**0.65 (0.59–0.79)**	0.75 (0.32–1.29)	
	100–200		**0.42 (0.15–0.57)**	**0.22 (0.13–0.71)**		**0.78 (0.51–0.83)**	0.74 (0.18–1.26)	
	200–350		**0.38 (0.20–0.59)**	**0.33 (0.14–0.35)**	p<0.01	**0.50 (0.28–0.76)**	0.56 (0.29–1.12)	p = 0.27
	350–500		**0.25 (0.10–0.46)**	**0.26 (0.18–0.52)**		**0.59 (0.48–0.72)**	0.49 (0.27–1.08)	
	>500		**0.54 (0.29–0.89)**	**0.42 (0.19–0.85)**		**0.54 (0.39–0.74)**	0.41 (0.31–1.09)	
Initiation year	2005		1.00	1.00		**1.00**	**1.00**	
	2006		**0.82 (0.54–0.97)**	**0.79 (0.48–0.96)**		0.62 (0.28–1.31)	0.73 (0.32–1.46)	
	2007		**0.75 (0.33–0.98)**	**0.70 (0.35–0.98)**	p = 0.01	**0.75 (0.19–0.92)**	**0.76 (0.28–0.93)**	p = 0.04
	2008		**0.72 (0.41–0.99)**	**0.68 (0.25–0.95)**		**0.86 (0.28–0.98)**	**0.82 (0.48–0.94)**	
	2009		**0.56 (0.43–0.72)**	**0.54 (0.41–0.80)**		**0.81 (0.31–0.88)**	**0.85 (0.32–0.91)**	
History of IDU^a^	No		1.00	1.00		1.00	1.00	
	Yes		**1.57 (1.18–1.88)**	**1.58 (1.31–1.78)**		**1.24 (1.08–1.54)**	**1.40 (1.25–1.89)**	
History of USS^b^	No		1.00	1.00		1.00	1.00	
	Yes		**0.62 (0.48–0.82)**	0.43 (0.24–1.27)		0.78 (0.45–1.07)	0.47 (0.78–1.47)	
Baseline BMI^c^ kg/m^2^			**0.96 (0.94–0.97)**	**0.96 (0.94–0.97)**		**0.98 (0.91–0.99**)	0.97 (0.88–1.03)	
Baseline OI^d^		No	1.00	1.00		1.00	1.00	
		Yes	**1.16 (1.02–1.45)**	1.29 (0.96–1.73)		1.47 (0.58–1.71)	1.72 (0.47–1.92)	
Baseline CTX^e^		No	1.00	1.00		1.00	1.00	
		Yes	**0.73 (0.48–0.85)**	0.43 (0.33–1.97)		0.97 (0.56–1.24)	0.47 (0.14–1.47)	
Baseline TB^f^		No	1.00	1.00		1.00	1.00	
		Yes	**1.35 (1.05–1.98)**	**1.61 (1.46–1.95)**		**1.32 (1.17–1.77)**	**1.28 (1.05–1.72)**	
Baseline WHO stage		Stage I	1.00	1.00		1.00	1.00	
		Stage II	2.61 (0.96–7.08)	1.90 (0.62–5.85)		1.64 (0.98–2.78)	1.50 (0.47–2.12)	
		Stage III	**7.88 (3.22–19.28)**	**4.55 (1.64–12.60)**	p = 0.05	2.77 (0.88–3.97)	2.13 (0.93–2.71)	p = 0.41
		Stage IV	**5.88 (2.41–14.30)**	**3.51 (1.26–9.77)**		3.47 (0.58–4.85)	3.10 (0.98–3.92)	
Adherence		Poor	1.00	1.00		1.00	1.00	
		Good	0.18 (0.14–1.88)	0.16 (0.06–1.28)		**0.48 (0.12–0.78)**	**0.55 (0.13–0.87)**	

*Note: Statistic significances were presented in bold.*

a *: IDU: Injecting drug user, ^b^: USS: Unsafe sex, ^c^: BMI [Body mass index], ^d^: OI: Opportunistic infection, ^e^: CTX:*

*Cotrimoxazol use, ^f^: TB: Tuberculosis*.

g *: HR is reported for one year increase in age*

### Associations between baseline characteristics and 6-month mortality

Table 3 shows that there is a strong association between year of initiation and the risk of six month mortality. After adjustment for other covariates, being enrolled in 2009 was significantly associated with a 46% lower risk of death within the first 6 months, compared with being initiated in 2005 (adjusted HR, 0.54, 95% CI 0.41–0.80). Also, having a baseline CD4 cell count of 200–350 cells/mm^3^ was significantly associated with a 67% lower risk of death in the first 6 months after ART initiation, compared with having a baseline CD4 count below 50 cells/mm^3^ (adjusted HR, 0.33, 95% CI 0.14–0.35).

The risk of six month mortality was significantly lower for females (adjusted HR, 0.54, 95% CI 0.34–0.85), but higher for being infected via injecting drugs (adjusted HR, 1.58, 95% CI 1.31–1.78), having TB at initiation (adjusted HR, 1.61, 95%CI 1.46–1.95), or being at a more advanced WHO clinical stage (adjusted HR stage IV compared to stage I, 3.51, 95%CI 1.26–9.77). Furthermore, for every unit increase in BMI, the risk of early mortality reduced by 4% (adjusted HR, 0.96, 95% CI 0.94–0.97).

Baseline age (for one year younger: adjusted HR 1.00, 95%CI 0.98–1.02), opportunistic infection (adjusted HR, 1.29, 95%CI 0.96–1.73), baseline Cotrimoxazol use (adjusted HR, 0.43, 95%CI 0.33–1.97), and adherence in the first six months (adjusted HR, 0.16, 95%CI 0.06–1.28) were not significantly associated with mortality in the first six months after ART initiation.

### Associations between patient characteristics and LTFU

In both univariate and multivariate models, younger patients were more likely to be LTFU than older patients (for one year younger: adjusted HR, 0.97, 95% CI 0.95–0.98). The risk of LTFU was significantly lower for females than for males (adjusted HR, 0.82, 95% CI 0.63–0.95), but was higher for those infected via IDU (adjusted HR, 1.40, 95% CI 1.25–1.89) and those who had TB at ART initiation (adjusted HR, 1.28, 95% CI 1.05–1.72). Being enrolled in 2009 was significantly associated with a lower risk of LTFU, compared with being enrolled in 2005 (adjusted HR, 0.85, 95% CI 0.32–0.91). Poor adherence was also significantly associated with LTFU in the same direction (adjusted HR, 0.55, 95% CI 0.13–0.87).

Baseline Cotrimoxazol use (adjusted HR, 0.47, 95%CI 0.14–1.47), baseline WHO clinical stage (adjusted HR stage IV versus stage 1, 3.10, 95%CI 0.98–3.92), baseline CD4 (adjusted HR 200–350 versus <50 cells/mm^3^, 0.56, 95%CI 0.28–1.12), and baseline BMI (for one unit increase: adjusted HR, 0.97, 95%CI 0.88–1.03) were not significantly associated with LTFU over the entire study period.

## Discussion

This study is the first published report to examine determinants of early mortality and loss to follow up among HIV patients receiving ART in Vietnam. In this well-defined cohort, we have identified patient characteristics that predict early mortality and LTFU in a resource-limited setting where a rapid scaling up of the ART programme is underway. Being male, having initiated ART in an earlier year, having a history of IDU and being co-infected with TB were all independently associated with a higher risk of both 6 month mortality and LTFU. Low baseline CD4 counts and BMI were significantly associated with early mortality, while being younger and having poor treatment adherence were predictive of LTFU. Baseline OI, baseline CTX did not identify patients at a higher risk for either of the two outcomes.

Our analysis showed a declining trend in the incidence of six month mortality and LTFU over the first five years of the ART programme in Vietnam. While the decreased trend in mortality rates was consistently observed in Africa [1], [6], [20], the reduced LTFU rates contrasts with the findings from South African studies [1], [20]. In conjunction with the rapid scaling up of ART treatment, the increased coverage of HIV-related services, improved quality of ART counselling [15], and less stringent eligible criteria to enrol patients with a less advanced HIV disease [21] could be key explanations for these positive outcomes. However, the six month mortality rate of 6% and the LTFU rate of 15% were still high, compared with other low- or middle-income countries where ART was implemented during the same period of time. LTFU in Myanmar, for example, was only 7% during this period [7].

Consistent with established evidence linking low baseline CD4 counts and higher risk of mortality, the present analysis showed that a low CD4 count level at ART initiation was a strong predictor of death within the first six months following treatment. Given 50% of patients initiated ART when their baseline CD4 cell count was below 50 cells/mm^3^, it is likely that the contribution of late ART initiation to mortality is substantial in the study cohort. Reasons previously identified for late initiation to ART in Vietnam and elsewhere include barriers to voluntary counselling and testing services, lack of routine CD4 cell count testing for newly diagnosed patients [22], long waiting time to enter programs, and stringent eligible criteria for ART initiation [21], [22]. As Vietnam has adopted WHO recommendations to initiate ART at a higher level of CD4 counts, special attention is required to strengthen post-test counselling as well as to improve CD4 count testing services, in order to ensure all patients have timely access to ART before the CD4 counts drop to a low level.

Positive associations between TB co-infection and early mortality have been consistently reported [9], [16]. Our observation of a strong relationship further confirms the role of TB in early mortality among Vietnamese HIV infected patients receiving ART. This is of particular importance as Vietnam has a high prevalence of TB and high proportions HIV-related deaths (e.g., of 40% [16]). Given that 33% of cohort participants were co-infected with TB at ART initiation, better integration of TB and HIV treatment could be usefully adopted at ART clinics. While TB- HIV integrated services have been suggested to reduce mortality and morbidity of both diseases in Vietnam [23], [24], this is yet to occur [25].

Poor adherence was an independent predictor for LTFU in our analysis, which replicates findings in other countries [26]–[28]. The absence of a statistically significant association between poor adherence and early mortality is also not without precedent: a Botswana study, for example, showed high rates of early mortality despite a high level of ART adherence [29]. One randomized control trial conducted in Quang Ninh province, Vietnam [16] also found no significant relationship between treatment adherence early mortality following ART initiation. The most likely explanation for these findings is that patients enrolled in these ART programs when their immunodeficiency was already too far advanced to protect them against early mortality, despite their good treatment adherence.

That 15.4% of patients were lost to follow up in the first 12 months of ART highlights that the patient tracking system in the study clinics could be improved. Two measures have been used to track patients through their clinical care since the inception of ART in Vietnam: telephone or text messages via mobile phone, and in-person field contact by peer educators or community-based support groups [30]. However, the implementation has not been without challenges. Available studies noted that home-based visits are not possible for the many patients who refuse to provide complete or accurate contact details, due to concerns over stigma and their confidentiality [30]. Also, many patients frequently change their residential location and thus cannot be easily tracked over time. Telephone or text message reminders are also difficult as many patients often change their number for the fear that their HIV status can be disclosed to their families or relatives [31]. To reduce LTFU, current patient tracking efforts will need critical assessment and special efforts are required to identify effective intervention strategies.

Our analysis indicated that patients with an IDU history were more likely than others to be lost to follow up. This was possibly because IDU participants feel that they have a double-stigma, not returning the ART clinic for continuing treatment. Furthermore, the presence of additional illness associated with injecting drugs may detract from the time allocated for ART services as there may be a need to attend other preventive and curative health services such as methadone treatment, needle exchanges, or treatment of other co-infections (i.e., hepatitis C, TB). More importantly, earlier studies in Vietnam and elsewhere [32] [33]–[35] noted that most HIV positive IDUs continue to inject drugs when they are on ART. Craving for drugs may inhibit their ability to adhere to their treatment schedule.

Younger age was the only demographic characteristic that predicted LTFU. This finding is consistent with a number of studies in Eastern, Southern, and Western Africa [36]–[38] [20], although other studies reported that older age is a significant predictor [8], [39] [7]. That young age is associated with LTFU may be explained by the fact that a large proportion of Vietnamese HIV patients receiving ART have an IDU history [40] and most IDUs are younger than non-IDU patients [32]. In the present analysis, there was statistic evidence indicating that the average age at ART initiation in the IDU group was significantly lower than the average age in the non-IDU group (28.7 versus 30.2, *p*<0.001) (data not shown).

Our study has several limitations. First, LTFU patients may have been identified simply on the basis of persistent absence from clinic appointments without tracing patients who have died without notification to the clinic. A recent study in South Africa, for example, found that 20–60% of patients LTFU have died during the follow up [41]. This shortcoming may have underestimated the true mortality rate among the cohort participants. To overcome this limitation, we used nomogram method [42] to correct mortality for LTFU, and the results are following: (i) Kaplan-Meier estimate for mortality of patients not lost to follow up: 5.74% (4.96, 6.52); (ii) Kaplan-Meier estimate for mortality of patients lost to follow up: 25% (16.6, 27.5); (iii) Estimate of overall mortality (%) for programme 8.7% (7.6, 10.0).

Second, data available for the present analysis were based on clinical records that were entirely dependent on the discretion of the responsible clinic staff leading to missing values in a number of variables. However, the missing values are likely to have minor impacts on the findings as they accounted for less than 5%. Further, as human resource shortages were reported in many clinics during the study period [22], it is possible that some patients may have been erroneously classified as LTFU by clinic staff who may already be overwhelmed with clinical duties.

Third, the definition of LTFU (no attendance within three months of the last scheduled visit) may have resulted in misclassification between treatment interruption and LTFU given some patients may have returned to a clinic after three months. Further analysis, however, showed that only 19 (0.6%) patients returned to a clinic more than three months after their last scheduled visit which clearly indicates that the impact of any such misclassification on the overall findings would be minimal. Similarly, the transfer of patients between ART clinics is unlikely to have resulted in them being misclassified as LTFU because all transfers require a referral letter from the old to the new clinic and details of the transfer must be recorded in patient records.

Fourth, the quality of the services provided by outpatient clinics, which may have a significant impact on LTFU and mortality, were not investigated in our study. Finally, the extent to which the findings are generalizable to all of Vietnam is unclear, given this study comprised 13 clinics from five provinces. Despite these shortcomings, a robust and consistent data protocol was adhered to which would minimize coding issues.

## Conclusion

Findings from this study suggest that the rapid expansion of the national ART programme in Vietnam has produced positive impacts. This was reflected in more patients being enrolled at earlier clinical stages with higher baseline CD4 counts, and decreased mortality and LTFU. However, the incidence of early mortality and LTFU remained significant and could benefit from further system and programmatic improvement. To reduce early mortality, special attention is required to ensure timely access to ART services. Loss to follow up also requires improvements after ART initiation. People who start ART with lower adherence, young people, and injecting drug users should be targeted through improving treatment counselling and the patient tracing system at ART clinics. Given that the number of HIV patients receiving ART in Vietnam will continue to increase resulting from the less stringent initiation criteria, additional studies are also needed to identify effective public health and medical interventions to reduce mortality and LTFU as well as to improve treatment outcomes, especially for the high-risk patient groups.
